# Body Mass Index, Waist-circumference and Cardiovascular Disease Risk Factors in Iranian Adults: Isfahan Healthy Heart Program

**DOI:** 10.3329/jhpn.v31i3.16831

**Published:** 2013-09

**Authors:** Noushin Mohammadifard, Masoud Nazem, Nizal Sarrafzadegan, Fatemeh Nouri, Firouzeh Sajjadi, Maryam Maghroun, Hassan Alikhasi

**Affiliations:** ^1^Isfahan Cardiovascular Research Center, Isfahan Cardiovascular Research Institute, Isfahan University of Medical Sciences, Isfahan, Iran; ^2^Medicine Faculty School, Isfahan Cardiovascular Research Institute, Isfahan University of Medical Sciences, Isfahan, Iran; ^3^Cardiac Rehabilitation Research Center, Isfahan Cardiovascular Research Institute, Isfahan University of Medical Sciences, Isfahan, Iran; ^4^Hypertension Research Center, Isfahan Cardiovascular Research Institute, Isfahan University of Medical Sciences, Isfahan, Iran

**Keywords:** Body mass index, Diabetes mellitus, Dyslipidaemia, Hypertension, Obesity, Risk Factor, Waist-circumference, Iran

## Abstract

Considering the main effect of obesity on chronic non-communicable diseases, this study was performed to assess the association between body mass index (BMI), waist-circumference (WC), cardiometabolic risk factors and to corroborate whether either or both BMI and WC are independently associated with the risk factors in a sample of Iranian adults. This cross-sectional study was performed on data from baseline survey of Isfahan Healthy Heart Program (IHHP). The study was done on 12,514 randomly-selected adults in Isfahan, Najafabad and Arak counties in 2000-2001. Ages of the subjects were recorded. Fasting blood glucose (FBG), 2-hour post-load glucose (2hpp), serum lipids, systolic and diastolic blood pressure (SBP and DBP), BMI, WC, smoking status, and total daily physical activity were determined. Increase in BMI and WC had a significant positive relation with the mean of FBG, 2hpp, SBP, DBP, serum lipids, except for HDL-C (p<0.001 for all). After adjustment for age, smoking, physical activity, socioeconomic status (SES), and BMI, the highest odds ratio (OR) (95% CI) for diabetes mellitus (DM) according to WC was 3.13 (1.93-5.08) and 1.99 (1.15-3.44) in women and men respectively. Moreover, the highest ORs based on BMI with adjustment for age, smoking, physical activity, SES, and WC were for dyslipidaemia (DLP) [1.97 (1.58-2.45) in women and 2.96 (2.41-3.63) in men]. The use of BMI or WC alone in the models caused to enhance all ORs. When both BMI and WC were entered in the model, the ORs for all risk factors, in men, according to BMI, were more compared to WC. However, in women, ORs for DM and hypertension (HTN) in WC quartiles were more than in BMI quartiles. BMI is the better predictor of DM, HTN, and DLP in men compared to WC. Conversely, in women, WC is a superior predictor than BMI, particularly for DM and HTN. Furthermore, the measurement of both WC and BMI in Iranian adults may be a better predictor of traditional risk factors of CVDs compared to BMI or WC alone.

## INTRODUCTION

Chronic non-communicable diseases (CNCDs), such as cardiovascular diseases (CVDs), cancers, and diabetes mellitus (DM), are the major causes of death worldwide (1). Moreover, CVDs are the leading cause of death in Iran (2) while the prevalence of coronary artery disease (CAD) was 19.4% in Isfahan, a central city of Iran (3).

Body mass index (BMI) has been identified as a potential of CVD risk factor for a long time (4−7). Central adiposity in adults make them more susceptible to major metabolic problems, such as ischaemic heart diseases, myocardial infarction, hypertension (HTN), dyslipidaemia (DLP), and dibetes mellitus (DM), and it cannot be indicated by BMI (8-10). It was assessed by measuring waist-circumference (WC) which is a better predictor than BMI for obesity outcomes (11,12). According to the National Institute of Health guidelines, it is supposed that BMI and WC have independent effects on obesity-related diseases (13). It is clear that combination of WC and BMI predicts health risks better than does BMI alone (9,10); however, the reverse is uncertain.

According to Adult Treatment Panel III (ATP III) criteria, about 33.8% of Iranian adults (more than 10.6 million) were centrally obese. It was about 4 times in females compared to males (14).

Considering the importance of CNCDs and the main effect of obesity on these diseases, this study was performed to assess the association between BMI, WC, and major cardiometabolic risk factors and to corroborate whether either or both BMI and WC are independently associated with these risk factors in a sample of Iranian adults who participated in the baseline survey of Isfahan Healthy Heart Program (IHHP). IHHP was a long-term community-based interventional programme for health promotion through reduction of CVD risk factors, and hence, reduction of morbidity and mortalitydue to CVDs. The study was conducted in 3 central counties of Iran (15).

## MATERIALS AND METHODS

### Sampling

This is an analytical study done with data of the baseline survey of IHHP (15,16). IHHP was undertaken in 3 counties of Isfahan, Najafabad, and Arak in the central part of Iran. According to the 2000 National Census, the population was 1,895,856, 275,084, and 668,531 in Isfahan, Najafabad, and Arak respectively (15,16). Multistage random-sampling technique was employed based on sex, age, and settlement distributions in each community to select 12,600 adults aged ≥19 years. Approximately 5-10% of households within these clusters were randomly selected. One individual aged ≥19 years per household was randomly selected. The selection criteria were: Iranian and mentally-competent individuals and also non-pregnant women (15,16).

Single eligible subject within the household was selected randomly from one of the six age-groups: 19-<25, 25-<35, 35-<45, 45-<55, 55-64, or 65 years and more (15).

### Data collection

Eligible individuals had a 30-minute home interview by trained health professionals. The questionnaire included questions on socioeconomic and demographic characteristics, health knowledge, cardiovascular risk-related attitudes and behaviours regarding dietary practice, smoking, and physical activity (15). Medical and drug history of participants were obtained for DLP, DM, and HTN by well-trained physicians.

Trained nurses obtained blood samples from the participants by venipuncture from the left antecubital vein after 12-14 hours of fasting. They kept all blood samples frozen at −20 °C to be assayed within 72 hours at the central laboratory of Isfahan Cardiovascular Research Center (ICRC) which meets the criteria of the National Reference Laboratory (a WHO-collaborating centre). Serum total cholesterol (TC) and triglyceride (TG) were determined by enzymatic method, using special kits (Immunodiagnostic, Germany) in an Elan 2000 auto-analyzer (Eppendorf, Germany). Also, HDL-C was measured by enzymatic method after precipitating the other lipoproteins with dextran sulphate magnesium chloride (17). LDL-C was calculated by using the Friedewald formula (18). Direct measurement of LDL-C was performed with a turbidimetric method for those with TG ≥400 mg/dL. Blood sugar, including fasting blood glucose (FBG) and 2-hour post-load plasma glucose (2hpp), were determined by glucose oxidase enzymatic method. To measure 2hpp, the blood sugar of non-diabetic participants was measured 2 hours after giving a syrup containing 75 g of glucose powder (19).

Physical measurements were done by trained medical staff with standardized methods. The weight was measured by a digital scale, with minimum necessary clothing, and recorded to the nearest 0.5 kg. Height was measured in a standing position, without shoes, to the nearest 0.5 cm, using a non-elastic stadiometer while the shoulders were in a normal state (20). BMI was calculated and recorded as weight in kg divided by height in metre squared (20). While the subjects were standing, WC and hip-circumference were measured by a tape at a level midway between the lower rib margin and iliac crest and at the point yielding the maximum circumference over the buttocks respectively (20). The blood pressure (BP) was measured by trained physicians based on standard criteria (21). These physicians were trained for a week to teach how to use sphygmomanometer and how to measure BP in a seated position. BP was measured two times in a seated position with a random-zero sphygmomanometer and an appropriate cutoff after a 5-minute rest. Average of the two measures was recorded for both systolic and diastolic BP (SBP and DBP).

### Ethics

Written informed consents were obtained from all participants, and the study was approved by the Ethical Committee of the ICRC (15).

### Definitions of risk factors and diseases

DLP was defined as having at least one of the following situations: TC ≥240 mg/dL and/or TG ≥200 mg/dL and/or LDL-C ≥160 mg/dL and/or HDL-C <40 mg/dL in men or HDL-C <50 mg/dL in women (22) and/or medications for hypolipidaemia. DM was defined as FBG ≥126 mg/dL and/or a 2hpp level of ≥200 mg/dL and/or medications for hypoglycaemia, using the WHO criteria (19). Individuals having SBP ≥140 mmHg and/or DBP ≥90 mmHg and/or using medications for BP were considered hypertensive patients (21).

### Statistical analysis

Statistical analyses were performed using SPSS for Windows software (version 15; SPSS, Chicago, IL, USA) and STATA software (version 10). The significance level was set at p value of <0.05. Comparison of the mean of variables with different quartiles of BMI and WC was done by analysis of covariance (ANCOVA), adjusting for the effect of age, smoking status (ever-smoker/non-smoker), total daily physical activity (METS per minute), and socioeconomic status (SES), including education and monthly income in each sex. The comparison of the mean value of BMI and WC in DM, HTN, and DLP patients who had either no other risk factors, one risk factor, or two risk factors were done by the analysis of variance (ANOVA). The chi-square test was used for comparing the prevalence of the risk factors in different quartiles of BMI and WC. The quantitative and qualitative basic characteristics were analyzed by *t*-test and chi-square test respectively.

Logistic regression analysis was also used for examining the independent and combined effects of BMI and WC on CVD risk factors. The odds ratios (ORs) in the 2^nd^, 3^rd^, and 4^th^ quartile of BMI and WC were compared with the 1^st^ quartile as reference. ORs were adjusted for the above potential confounding variables. BMI and WC were entered into the regression model as continuous variables.

Preliminarily, the models with and without interaction effect were compared by likelihood ratio test (LR), Akaike Information Criterion (AIC), and Bayesian Information Criterion (BIC) tests. The results of these analyses confirmed that the combined model with BMI and WC should be reported without their interaction effect.

To test for linear trend of ORs and determine p for trend across quartile of BMI or WC, we assigned the median BMI or WC to varying individuals as continuous variables in logistic regression for hypertensive vs non-hypertensive, diabetic vs non-diabetic hyperlipidaemic vs normolipidaemic subjects.

## RESULTS

The study participants comprised 12,514 adults. We limited the current analysis to 12,416 individuals, including 6,081 men and 6,335 women because we did not have enough data for 98 individuals to be included in this analysis. The basic characteristics of the participants are presented in Table 1.

The adjusted mean values of BMI, WHR, FBG, 2hpp, TC, serum lipids, and BPs in different quartiles of WC based on sex are shown in Table 2. Increase in WC had significant positive relationship with increase in the mean of BMI, WHR, FBG, 2hpp, TC, TG, LDL-C, SBP, and DBP but with decrease in HDL-C (p<0.001 for all). Comparison of DM, HTN, and HLP prevalence in WC quartiles according to sex is presented in Table 3. The DM, HTN, and DLP prevalence had a significant relationship with increase in WC (p<0.001 for all). Table 4 indicates that rise in BMI had a significant direct association with increase in the mean adjusted values of WC, WHR, FBG, 2hpp, TC, TG, LDL-C, SBP, and DBP and decrease in HDL-C. DM, HTN and DLP prevalence had a significant relationship with increase in BMI (p<0.001 for all) (Table 5). Adjusted ORs for HTN, DLP, and DM according to WC and BMI quartiles are presented in Table 6.

When both BMI and WC were entered in the model, the ORs for all risk factors, in men, according to BMI were comparable to WC. Moreover, ORs for DLP based on BMI were more than on WC in both the sexes. Conversely to men, ORs for DM and HTN in WC quartiles were more compared to BMI quartiles in women. The trends in ORs for all risk factors were gradually increased significantly by enhancing both BMI and WC quartiles (p for trend <0.05 for all). The use of BMI or WC alone in the models caused to enhance all ORs (data not shown).

**Figure 1.**
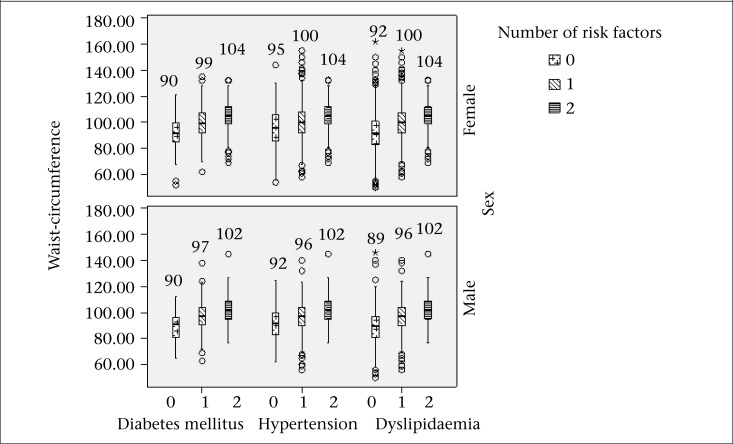
The mean of waist-circumference in diabetic, hypertensive and dyslipidaemic subjects with different numbers of risk factors based on sex

**Figure 2.**
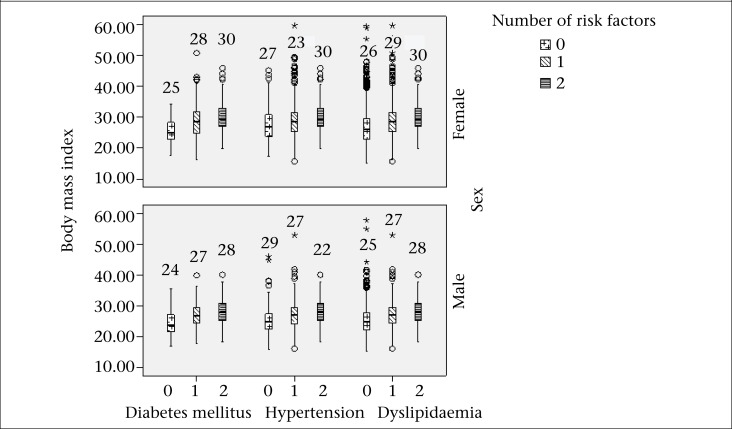
The mean of body mass index in diabetic, hypertensive and dyslipidaemic subjects with different numbers of risk factors based on sex

In total population, the mean values of WC significantly increased [90.6±14.4, 98±12.2, and 103.5±11.6 (p for trend <0.001)] in those with DM and no other risk factor, with one risk factor, and with two risk factors respectively. These were 93.2±13.7, 98±13.3, and 103.6±11.6 respectively in hypertensive subjects (p for trend <0.001). In dyslipidaemic subjects, the mean level of WC were 90.5±12.7, 98.1±13.2, and 103.6±11.6 respectively (p for trend <0.001). BMI (mean±SD) in diabetes and no other risk factor, with one risk factor, and with two risk factors were 24.5±4.2, 27.6±4.7, and 29.4±4.5 respectively (p for trend <0.001). These were 27.8±4.5, 24.9±5.1, and 26.4±4.5 in hypertension respectively (p for trend <0.001). In dyslipidaemic subjects, BMI (mean±SD) was 25.7±4.6, 27.9±5, and 29.4±4.5 respectively (p for trend <0.001). Morevover, the mean value of WC and BMI in diabetic, hypertensive and dyslipidaemic subjects with different numbers of CVD risk factors based on sex are illustrated in Figure 1 and 2. All values were significant (p<0.001).

**Table 1.** General characteristics of study participants based on gender

Variable	Total (n=12,416)	Male (n=6,081)	Female (n=6,335)
	Mean±SD	Mean±SD	Mean±SD
Age (years)	38.9±14.9	39.0±15.3	38.8±14.5
Body mass index (kg/m^2^)	25.6± 4.8	24.5±4.2	26.6±5.2
Waist-circumference (cm)	90.5±13.3	88.4±12.0	92.6±14.1
Waist-to-hip ratio	0.91±0.09	0.9±0.08	0.90±0.09
Fasting blood glucose (mg/dL)	83.8±31.8	83.5±29.9	84±33.5
Glucose (2hpp) (mg/dL)	101.3±50.9	96.9±47.4	105.6±53.6
Total cholesterol (mg/dL)	198.2±50.0	194.0±49.4	202.3±50.3
Triglycerides (mg/dL)	170.0±110.3	178.5±120	161.9±99.5
HDL-C (mg/dL)	46.8±10.7	45.3±10.5	48.3±10.6
LDL-C (mg/dL)	118.2±41.3	114.2±40.6	122.1±41.6
Systolic blood pressure (mmHg)	115.9±19.4	116.6±18.2	115.2±20.6
Diastolic blood pressure (mmHg)	75.6±11.3	75.9±10.7	75.3±11.9
Prevalence (%)			
Diabetes mellitus	5.6	5	6.3
Hypertension	17.3	15.6	18.9
Dyslipidaemia	76.6	69.1	83.7

SD=Standard deviation

## DISCUSSION

The results indicated that higher BMI and WC were significantly associated with HTN, DLP, and DM. Adjusted odds ratio of CVD risk factors by age, smoking, SES, and total daily physical activity showed that the occurrence of DM, HTN, and DLP is significantly related with increase in obesity indicators. As the odds ratio in the combined models with BMI and WC were less than that in the models with BMI or WC alone, we concluded that assessing both BMI and WC may be a better predictor of CVD risk factors compared to BMI or WC alone. Ying *et al*. reported similar results in young and middle-aged Chinese women (23).

Our results are in conformity to similar studies in Korea, India, Australia, and Singapore. In these studies, the incidence of DM and HTN was found to increase with higher levels of BMI even in those whose BMI was within normal range (10,24-27).

A study in Japan performed by Ito and his colleagues showed that the risk of DLP, including high levels of LDL-C, and TG was significantly more in those placed in the highest one-third of WC compared to those placed in the lowest one-third of this index (28). Our study illustrated that the risk of DLP is significantly higher in the highest quartile of obesity indicators, especially BMI in both the sexes. In general, there was a positive significant relationship between the quartiles and the mean of serum lipids and the prevalence of DLP. In another study done by Tanaka and his colleagues in Japan, the same finding was reported in such a way that individuals with WC in the upper quartile had significantly higher prevalence of CVD risk factors. This study noted that at least one of the CVD risk factors increased with higher levels of WC (29).

Obesity may cause insulin resistance, leading to DM, HTN, and DLP (26-28). These results can be confirmed by significant relationship between BMI and WC, and these CVD risk factors are presented in our study.

**Table 2.** Comparing the adjusted* mean value of cardiovascular risk factors in waist-circumference quartiles based on gender

Variable	Female				Male			
	1^st^ quartile WC ≤83	2^nd^ quartile 83 <WC ≤93	3^rd^ quartile 93 <WC ≤102	4^th^ quartile WC >102	1^st^ quartile WC ≤80	2^nd^ quartile 80 <WC ≤88	3^rd^ quartile 88 <WC ≤97	4^th^ quartile WC> 97
Body mass index (kg/m^2^)	22.35 (21.99-22.69)	25.17 (24.83-25.51)	27.75 (27.41-28.09)	31.67 (31.32-32.01)	21.06 (20.91-21.21)	23.19 (23.03-23.35)	25.46 (25.31-25.62)	28.85 (28.68-29.01)
Waist-to-hip ratio	0.825 (0.818-0.831)	0.887 (0.881-0.893)	0.929 (0.923-0.935)	0.977 (0.970-0.983)	0.840 (0.837-0.843)	0.885 (0.882-0.888)	0.916 (0.913-0.919)	0.964 (0.960-0.967)
Fasting blood glucose (mg/dL)	79.74 (77.48-82.00)	80.67 (78.46-82.89)	82.71 (80.49-84.94)	86.27 (84.03-88.51)	80.73 (79.46-82.00)	80.81 (79.45-82.19)	84.57 (83.26-85.87)	86.99 (85.57-88.41)
Glucose (2hpp) (mg/dL)	98.03 (93.64-102.42)	99.62 (95.32-103.92)	101.81 (97.49-106.13)	108.88 (104.52-113.24)	92.59 (90.45-94.74)	91.77 (89.45-94.09)	97.64 (95.43-99.85)	103.71 (101.29-106.13)
Total cholesterol (mg/dL)	189.59 (185.49-193.67)	198.15 (194.15-202.15)	205.34 (201.3-209.36)	216.16 (212.11-220.21)	180.76 (178.49-183.03)	189.79 (187.34-192.23)	199.48 (197.15-201.81)	207.88 (205.35-210.41)
Triglyceride (mg/dL)	139.88 (131.51-148.25)	156.18 (147.99-164.37)	180.63 (172.39-188.86)	207.10 (198.81-215.39)	132.18 (126.57-137.79)	160.93 (154.85-166.99)	201.77 (196-207.55)	232.92 (226.65-239.19)
HDL-C (mg/dL)	48.95 (47.99-49.91)	48.27 (47.33-49.20)	47.15 (46.21-48.09)	47.91 (46.96-48.86)	45.74 (45.25-46.23)	45.27 (44.74-45.79)	44.89 (44.39-45.40)	44.06 (43.51-44.60)
LDL-C (mg/dL)	115.16 (111.48-118.85)	120.61 (117.01-124.21)	124.93 (121.29-128.56)	130.37 (126.69-134.03)	109.42 (107.45-111.39)	113.39 (111.24-115.53)	116.03 (113.96-118.11)	120.12 (117.83-122.41)
Systolic blood pressure (mmHg)	109.55 (107.99-111.12)	111.42 (109.89-112.96)	114.71 (113.17-116.25)	118.91 (117.36-120.46)	112.42 (111.65-113.19)	114.36 (113.52-115.19)	116.89 (116.1-117.69)	120.69 (119.83-121.57)
Diastolic blood pressure (mmHg)	72.57 (71.61−73.54)	73.03 (72.09−73.98)	75.08 (74.13−76.03)	77.37 (76.42−78.33)	73.85 (73.37−74.33)	74.26 (73.75−74.78)	76.07 (75.58−76.57)	78.49 (77.96−79.03)

*Adjusted for age, smoking status, total daily physical activity, and socioeconomic status;

All p values are less than 0.001; Figures in parentheses are 95% confidence intervals

**Table 3.** Comparing the prevalence of cardiovascular risk factors in waist-circumference quartiles based on gender

Variable	Female				Male			
	1^st^ quartile WC ≤83	2^nd^ quartile 83 <WC ≤93	3^rd^ quartile 93 <WC ≤102	4^th^ quartile WC >102	1^st^ quartile WC ≤80	2^nd^ quartile 80 <WC ≤88	3^rd^ quartile 88 <WC ≤97	4^th^ quartile WC >97
Diabetes mellitus	30 (1.8%)	72 (4.4%)	116 (7.5%)	176 (11.9%)	26 (1.5%)	30 (2.2%)	92 (6.0%)	146 (10.5%)
Hypertension	124 (7.4%)	218 (13.3%)	348 (22.4%)	504 (33.9%)	115 (6.7%)	140 (10.0%)	268 (17.3%)	427 (30.4%)
Dyslipidaemia	1,045 (63.3%)	1,143 (70.0%)	1,226 (79.4%)	1,229 (83.2%)	669 (39.5%)	707 (50.9%)	958 (62.5%)	1,020 (73.1%)

All p values are less than 0.001

**Table 4.** Comparing the adjusted* mean values of cardiovascular risk factors in body mass index quartiles based on gender

Variable	Female				Male			
	1^st^ quartile BMI ≤22.77	2^nd^ quartile 22.77 <BMI ≤26.17	3^rd^ quartile 26.17 <BMI ≤29.72	4^th^ quartile BMI >29.72	1^st^ quartile BMI ≤21.39	2^nd^ quartile 21.39 <BMI ≤24.16	3^rd^ quartile 24.16 <BMI ≤27.14	4^th^ quartile BMI >27.14
Waist-circumference (cm)	81.44 (80.53-82.36)	89.69 (88.79-90.59)	96.58 (95.67-97.48)	105.90 (105.01-106.79)	78.57 (78.15−78.99)	84.24 (83.83-84.65)	90.93 (90.52-91.35)	99.49 (99.07-99.90)
Waist-to-hip ratio	0.875 (0.867-883)	0.900 (0.892-907)	0.917 (0.909-.924)	0.934 (0.926-0.941)	0.861 (0.858-0.864)	0.885 (0.882-0.888)	0.908 (0.905-0.911)	0.938 (0.934-.941)
Fasting blood glucose (mg/dL)	79.17 (76.89-81.44)	81.43 (79.20-83.66)	82.52 (80.29-84.76)	85.60 (83.38-87.82)	80.85 (79.52-82.19)	81.93 (80.62-83.23)	82.78 (81.45-84.10)	87.08 (85.75-88.41)
Glucose (2hpp) (mg/dL)	96.44 (92.10-100.83)	101.68 (97.34-106.00)	101.40 (97.05-105.75)	107.74 (103.43-112.06)	93.20 (90.95-95.46)	92.73 (90.51-94.95)	95.17 (92.93-97.42)	103.85 (101.58-106.11)
Total cholesterol (mg/dL)	187.96 (183.88-192.1)	199.72 (195.71-203.73)	207.31 (203.28-211.33)	214.87 (210.86-218.87)	177.58 (175.23-179.9)	189.16 (186.84-191.47)	200.57 (198.23-202.92)	208.97 (206.62-211.33)
Triglyceride (mg/dL)	137.30 (128.89-145.70)	160.62 (152.38-168.8)	180.29 (172.02-188.58)	202.05 (193.82-210.28)	127.47 (121.65-133.28)	156.56 (150.84-162.29)	201.37 (195.56-207.17)	235.67 (229.85-241.50)
HDL-C (mg/dL)	49.18 (48.22-50.14)	48.49 (47.55-49.4)	47.67 (46.73-48.61)	47.18 (46.24-48.11)	46.13 (45.62-46.65)	45.05 (44.54-45.55)	44.67 (44.16-45.18)	44.28 (43.77-44.79)
LDL-C (mg/dL)	113.28 (109.6-116.96)	121.89 (118.28-125.51)	126.19 (122.56-129.83)	129.49 (125.86-133.12)	106.90 (104.86-108.95)	113.32 (111.29-115.36)	118.41 (116.30-120.52)	120.06 (117.91-122.20)
Systolic blood pressure (mmHg)	110.01 (108.4-111.6)	111.68 (110.14-113.23)	113.85 (112.29-115.40)	118.07 (116.53-119.61)	112.82 (112-113.63)	114.39 (113.59-115.20)	116.37 (115.56-117.18)	120.09 (119.28-120.91)
Diastolic blood pressure (mmHg)	71.76 (70.8−72.72)	73.19 (72.25−74.13)	74.80 (73.86−75.75)	77.55 (76.61−78.48)	73.19 (72.69−73.69)	74.89 (74.39−75.39)	75.99 (75.49−76.49)	78.29 (77.79−78.79)

*Adjusted for age, smoking status, daily physical activity, and socioeconomic status;

All p values are less than 0.001; Figures in parentheses are 95% confidence intervals

**Table 5.** Comparing the prevalence of cardiovascular risk factors in body mass index quartiles based on gender

Variable	Female				Male			
	1^st^ quartile BMI ≤22.77	2^nd^ quartile 22.77 <BMI ≤26.17	3^rd^ quartile 26.17 <BMI ≤29.72	4^th^ quartile BMI >29.72	1^st^ quartile BMI ≤21.39	2^nd^ quartile 21.39 <BMI ≤24.16	3^rd^ quartile 24.16 <BMI ≤27.14	4^th^ quartile BMI >27.14
Diabetes mellitus	35 (2.2%)	85 (5.4%)	115 (7.2%)	158 (10.1%)	24 (1.6%)	47 (3.1%)	81 (5.4%)	141 (9.4%)
Hypertension	134 (8.5%)	231 (14.5%)	346 (21.7%)	478 (30.4%)	108 (7.1%)	172 (11.4%)	262 (17.2%)	407 (26.8%)
Dyslipidaemia	962 (61.5%)	1,120 (71.2%)	1,244 (78.3%)	1,300 (83.2%)	555 (37.0%)	748 (49.8%)	945 (62.8%)	1,102 (73.4%)

All p values are less than 0.001

**Table 6.** Adjusted* odds ratio (95% CI) of cardiovascular risk factors in waist-circumference and body mass index quartiles in comparison with 1^st^ quartile

Risk factor		Waist-circumference					Body mass index				
		1^st^ quartile	2^nd^ quartile	3^rd^ quartile	4^th^ quartile	p for trend	1^st^ quartile	2^nd^ quartile	3^rd^ quartile	4^th^ quartile	p for trend
Female	Diabetes mellitus	1	1.71 (1.07-2.71)	2.29 (1.44-3.65)	3.13 (1.93-5.08)	<0.001	1	1.39 (0.89-2.15)	1.38 (0.88-2.17)	1.67 (1.05-2.66)	0.041
	Hypertension	1	1.31 (0.99-1.72)	1.81 (1.37-2.39)	2.43 (1.81-3.28)	<0.001	1	1.03 (0.78-1.35)	1.27 (0.96-1.68)	1.76 (1.31-2.35)	<0.001
	Dyslipidaemia	1	1.06 (0.90-1.24)	1.39 (1.15-1.68)	1.41 (1.12-1.77)	0.001	1	1.29 (1.09-1.51)	1.61 (1.33-1.94)	1.97 (1.58-2.45)	<0.001
Male	Diabetes mellitus	1	0.95 (0.54-1.68)	1.84 (1.09-3.08)	1.99 (1.15-3.44)	0.004	1	1.35 (0.79-2.30)	1.68 (0.98-2.90)	2.67 (1.52-4.68)	<0.001
	Hypertension	1	1.07 (0.80-1.44)	1.38 (1.03-1.85)	1.74 (1.27-2.38)	<0.001	1	1.19 (0.89-1.59)	1.46 (0.97-1.98)	2.30 (1.67-3.17)	<0.001
	Dyslipidaemia	1	1.25 (1.07-1.46)	1.54 (1.29-1.84)	1.94 (1.57-2.40)	<0.001	1	1.47 (1.26-1.72)	2.12 (1.78-2.53)	2.96 (2.41-3.63)	<0.001

*Adjusted for age, smoking status, daily physical activity, and socioeconomic status and combined model with waist-circumference and body mass index;

Figures in parentheses are (95% confidence intervals

Conversely, several studies demonstrated that WC or other central obesity indicators might be superior predictors of DLP (30-32). Furthermore, according to the multiple logistic regression analysis reported herein, WC in women and BMI in men were the better predictor of DM and HTN. It may occur since the cut-points of central obesity in men based on both ATP III and International Diabetes Federation (IDF) criteria (22,33) were higher than the 1^st^ quartile of WC, which was considered the reference value to estimate the ORs in this study. However, various studies had different results. In some of these, WC was a better and accurate measure of CVD risk (16-18). For instance, Vazquez *et al.* reported that abdominal obesity was a stronger predictor of DM incidence than overall obesity (34). However, in the US and European Caucasians, overall obesity was a better predictor of DM (35). Also, Knowles *et al.* noted that WC was the best predictor of HTN in men and DM in women (36). In several studies, WC had a much stronger relationship with DM, HTN, and DLP (37-39) while in some others, both WC and BMI were worthy predictors of the CVD risk factors (11,40-41). Furthermore, a meta-analysis illustrated that BMI and WC were equally good in forecasting DM (34). Thus, measurement of both BMI and WC can improve CVD risk stratification (11).

In this study, along with the other study performed by Lee *et al.* on the Chinese living in Hong Kong (42), the mean BMI and WC were higher in DM, HTN and DLP subjects, with the presence of greater number of risk factors, which can demonstrate the increasing effect of both kinds of obesity indices in causing multiple risk factors.

### Limitations

The use of cross-sectional data to determine the relationship between anthropometric indices and CVD risk factors is the limitation of this research work. Therefore, we were not exactly able to analyze the causal relationship between obesity and the risk factors in this study.

### Conclusions

We conclude that BMI is the better predictor of DM, HTN, and DLP in men compared to WC. Conversely in women, WC is a superior predictor than BMI, particularly for DM and HTN subjects. So, BMI alone might be a useful indicator in Iranian men if measuring WC is difficult. Furthermore, the measurement of both WC and BMI in Iranian adults may be a better predictor of traditional risk factors of CVDs than BMI or WC alone. Therefore, it is suggested that both BMI and WC be regular measures for identification of the high-risk obese population in epidemiological studies.

## ACKNOWLEDGEMENTS

This study was conducted by the ICRC (a WHO-collaborating centre) in collaboration with Isfahan Provincial Health Office, both of which are affiliated with the Isfahan University of Medical Sciences. The work was supported by a grant (No. 31309304) from the Iranian Budget and Planning Organization as well as the Deputy for Health of the Iranian Ministry of Health and Medical Education and the Iranian Heart Foundation. We are thankful to the team of ICRC and Isfahan Provincial Health Office as well as collaborators from Najafabad Health Office and Arak University of Medical Sciences.
